# Upper and lower gastrointestinal endoscopies in patients over 85 years of age: Risk-benefit evaluation of a longitudinal cohort

**DOI:** 10.1097/MD.0000000000008439

**Published:** 2017-11-03

**Authors:** Raphaël Clere-Jehl, Mickael Schaeffer, Thomas Vogel, Michele Kiesmann, Jean-Louis Pasquali, Emmanuel Andres, Anne Bourgarit, Bernard Goichot

**Affiliations:** a Internal Medicine, Endocrinology and Nutrition Department, Hautepierre Hospital; b Medical Information and statistics Department, Civil Hospital; c Geriatric Department, Robertsau Hospital; d Internal Medicine Department, New Civil Hospital; e Internal Medicine Department, Civil Hospital, University Hospital of Strasbourg, Strasbourg, France.

**Keywords:** complications, diagnosis, elderly, gastrointestinal endoscopy, indications, iron deficiency anemia, outcome

## Abstract

After age 85, upper and lower gastrointestinal (GI) endoscopy may be indicated in 5% to 10% of inpatients, but the risk–benefit ratio is unknown. We studied patients older than 85 years undergoing upper and lower GI endoscopy.

We analyzed a retrospective cohort of inpatients older than 85 years between 2004 and 2012, all explored by upper and complete lower GI endoscopy. Initial indications, including iron deficiency anemia (IDA), other anemias, GI bleeding, weight loss, and GI symptoms, were noted, as were endoscopy or anesthesia complications, immediate endoscopic diagnosis, and the ability to modify the patients’ therapeutics. Deaths and final diagnosis for initial endoscopic indication were analyzed after at least 12 months.

We included 55 patients, 78% women, with a median age, reticulocyte count, hemoglobin, and ferritin levels of 87 (85–99), 56 (24–214) g/L, 8.6 (4.8–12.9) g/dL, and 56 (3–799) μg/L, respectively. IDA was the most frequent indication for endoscopy (60%; n = 33). Immediate diagnoses were found in 64% of the patients (n = 35), including 25% with GI cancers (n = 14) and 22% with gastroduodenal ulcers or erosions (n = 12). Cancer diagnosis was associated with lower reticulocyte count (45 vs. 60 G/L; *P* *=* .02). Among the 35 diagnoses, 94% (n = 33) led to modifications of the patients’ therapeutics, with 29% of the patients deciding on palliative care (n = 10). No endoscopic complications lead to death. Follow-up of >12 months was available in 82% (n = 45) of the patients; among these patients, 40% (n = 27) died after an average 24 ± 18 months. Cancer diagnosis was significantly associated with less ulterior red cell transfusion (0% vs. 28%; *P* *=* .02) and fewer further investigations (6.7% vs. 40%; *P* = .02).

Upper and complete lower GI endoscopy in patients older than 85 years appears to be safe, and enables a high rate of immediate diagnosis, with significant modifications of therapeutics. GI cancers represented more than one-third of the endoscopic diagnoses.

## 1 Introduction

Upper and lower gastrointestinal (GI) endoscopies frequently have a theoretical indication in elderly patients. Indeed, iron deficiency anemia (IDA) is an indication for GI endoscopies^[1–5]^ in 5% to 10% of internal medicine inpatients older than 85 years. The prevalence of anemia reaches 25% among geriatric populations,^[6–8]^ and 20% to 30% of anemia cases are found to be owing to iron deficiency in patients older than 65 years.^[9]^ Weight loss and isolated GI symptoms are additional indications for GI endoscopy.^[10–12]^

However, upper and complete lower GI endoscopies are invasive procedures requiring anesthesia,^[13]^ which deters physicians from performing GI endoscopies in elderly patients. Moreover, older age and comorbidities may prevent physicians from acting on an endoscopic diagnosis, making clinical benefit uncertain.

On the other side, GI endoscopy may be useful for diagnoses in elderly patients.^[14]^ Upper and lower GI endoscopies enable a causal diagnosis in 63% to 85% of patients with IDA^[15–17]^ ; this rate appears to be higher in patients older than 65 years, reaching 90%^[18]^ and including 9% to 16% as GI cancers.^[15,16,19,20]^

No risk–benefit study of GI endoscopies in patients older than 85 years of age is available. To address this clinical issue, we evaluated the initial indications (IDA and others), findings, and complications of upper and lower GI endoscopies in patients older than 85 years of age. We also evaluated clinical outcome at least one year after the endoscopic procedure.

## 2 Methods

### 2.1 Patients

All in-patients older than 85 years of age who had undergone upper and lower GI endoscopy between January 2004 and December 2012 in 4 different internal medicine units of Strasbourg's University Hospital (France) were prospectively registered and retrospectively considered. To be included, patients were required to have had both upper and complete lower GI endoscopy. Our longitudinal retrospective cohort study received the approval of the ethics committee of the Faculty of Medicine at the University of Strasbourg in April 2015.

### 2.2 Baseline characteristics

The patients’ epidemiologic characteristics and any treatment that could potentially induce bleeding, that is, the use of nonsteroidal anti-inflammatory drugs (NSAIDs), antiplatelets, and anticoagulants, were recorded. Hemoglobin (Hb) levels, mean corpuscular volume (MCV), reticulocyte counts, serum ferritin levels, transferrin saturation, folic acid, and vitamin B12 deficiencies were recorded at inclusion.

Data about anesthesia were collected, including the patients’ American Society of Anesthesiologists (ASA) physical status, as well as the type of anesthetic drugs and techniques used.

Initial indications of GI endoscopies were recorded; each patient could have ≥1 indications. Anemia was defined by hemoglobin <13 g/dL among men and 12 g/dL among women, and iron deficiency anemia (IDA) was defined by serum ferritin level <100 μg/L concomitant with anemia. Other indications were GI bleeding, weight loss in excess of 10% in <6 months, GI symptoms, and cancers of unknown primary origin.

### 2.3 GI endoscopies: results and complications

Significant GI lesions diagnosed by initial GI endoscopy were recorded, including cancers of any part of the explored bowels (esophagus, stomach, colon, and rectum), ulcers or erosions, esophagitis, angiodysplasias, ischemic colitis, and polyps >10 mm.

In cases of anemia without significant GI lesion, minimal digestive lesions not considered to be the cause of anemia alone were noted (i.e., hiatal hernia, colonic diverticulosis, nonerosive gastritis, haemorrhoidal diseases, colonic polyps <10 mm); when these minimal digestive lesions were associated with antiplatelets, anticoagulants, or NSAIDs, this association was recorded.

Each therapeutic modification made as a result of GI endoscopy was noted, including the decision for palliative treatment.

Complications owing to GI endoscopy or anesthesia were noted, including death, life-threatening complications, and mild complications. If present, colon perforations and cardiovascular events were considered life-threatening complications, whereas delirium following anesthesia was classified as a mild complication.

### 2.4 Patient follow-up

When information was available in medical records, a minimal 12-month follow-up was considered sufficient.

Information about outcome was collected in 2016 by analyzing data of new in-patient stays or consultations and by phone call to the general practitioner of the patient. The following were recorded: survival, new diagnosis as a result of initial GI endoscopy indication, causes of death (and any connection with the indication of GI endoscopy), persistence of anemia, and red cell transfusions. Investigations for diagnostic purposes, performed after initial GI endoscopy, were called *“*further investigations”.

### 2.5 Statistical analyses

Quantitative variables were described using position and dispersion statistics as the mean and standard deviation. Qualitative variables were described by effectives and percentages. Comparisons between quantitative variables were accomplished with Student *t* test or nonparametric Mann–Whitney *U* test. To compare qualitative variables, the nonparametric Fisher exact test was used. The significance level was fixed at 5%. Analyses were performed with the R software (Language for Environment and Statistical computing, R Core team, Vienna, Austria) under its version 3.1 with all additional packages required.

## 3 Results

### 3.1 Inclusion flow chart

**Figure 1:**
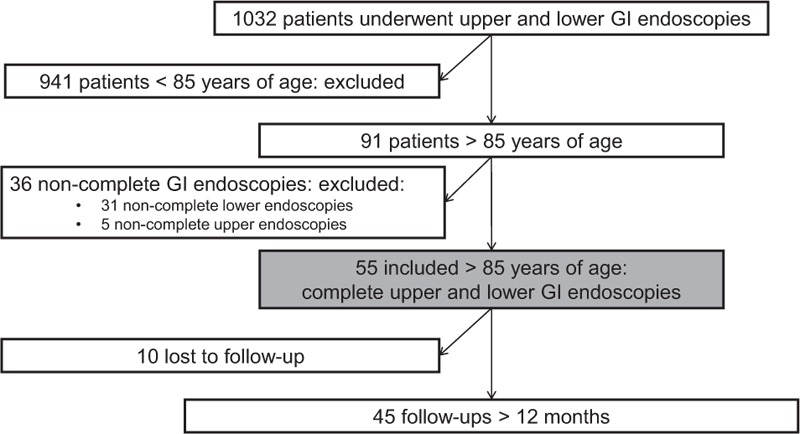
Flow chart. GI = gastrointestinal, IDA = iron deficiency anemia.

Among 1032 internal medicine inpatients who underwent a GI endoscopic procedure (the inclusion flow chart is shown in Fig. 1), 8.8% (n = 91) were at least 85 years of age. Of these 91 patients, 60% (n = 55) had complete upper and lower GI endoscopy and were included.

A follow-up of at least 12 months was finally obtained for 82% (n = 45) of the included patients.

### 3.2 Baseline features of patients

At baseline (Table 1), these 55 patients were 88.0 ± 2.0 years’ old; 18% lived in nursing homes, and 78% were women. The patients had a median hemoglobin level, MCV, reticulocyte count, and serum ferritin level of 8.6 ± 2.1 g/dL, 85 ± 10 μm^3^, 59 ± 21 g/L and 112 ± 261 μg/L, respectively. The patients presented with folic acid deficiency in 29% (n = 16) of cases and vitamin B12 deficiency in 22% (n = 12) of cases. Seventy-seven percent of the patients (n = 42) suffered from cardiovascular diseases, among them 51% (n = 28) had chronic heart failure. Sixty-seven percent (n = 37) were treated with at least 1 antithrombotic therapy: Vitamin K antagonist in 26% (n = 14), antiplatelets in 40% (n = 22), and NSAID in 7.3% (n = 4).

**Table 1:**
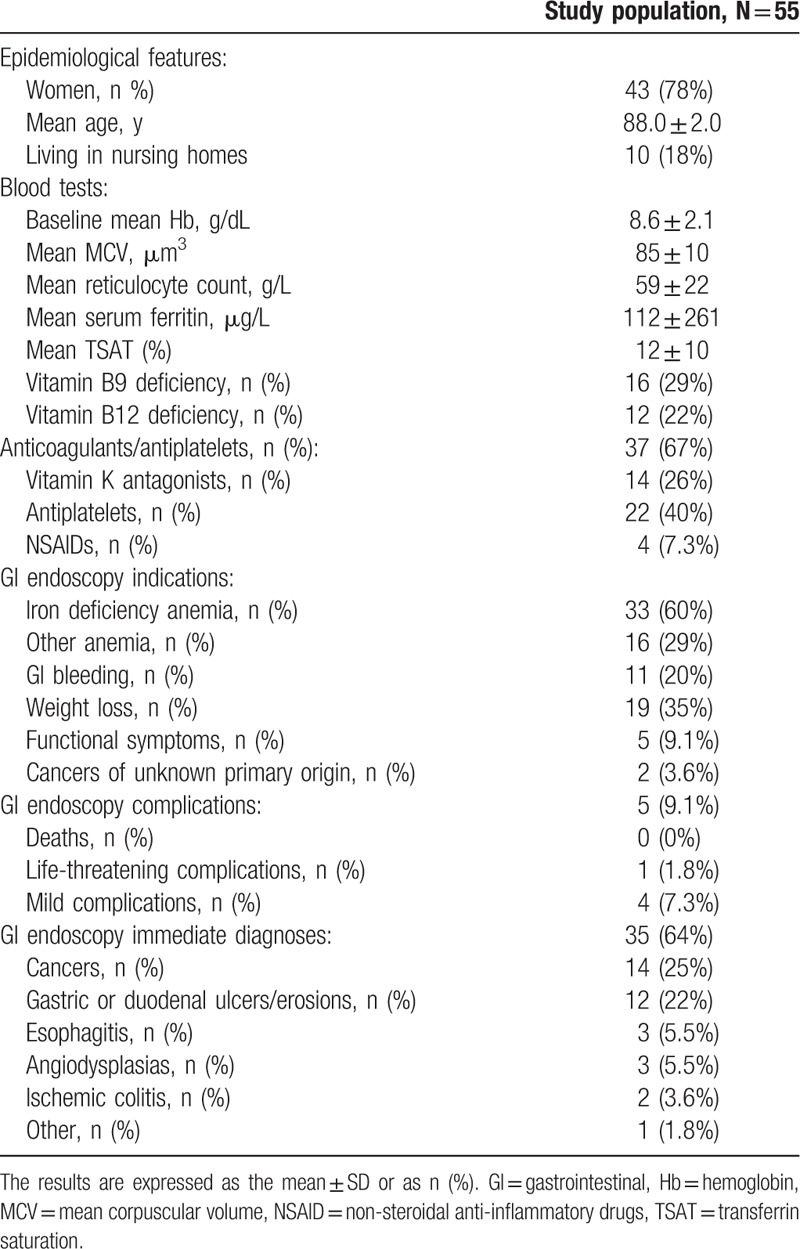
Patients characteristics at baseline.

### 3.3 Anesthesia

All patients had an ASA status of 2 (35%, n = 19) or 3 (65%, n = 36). Over half of ASA scores of 3 were because of chronic heart failure or coronary heart disease. All patients underwent a general anesthesia without intubation, and all included patients had a colonoscopy preparation for elective upper and complete lower GI endoscopies. Anesthetic drugs were propofol in 95% of the patients (n = 52), ketamine chlorhydrate in 3% (n = 2), or both in 2% (n = 1). Doses of anesthetic drugs were reduced by 50% on average compared with younger adult patients.

### 3.4 Indications of GI endoscopy

IDA was the most frequent indication for GI endoscopy, at 60% (n = 33), followed by weight loss in 35% (n = 19), other anemia in 29% (n = 16), and GI bleeding in 20% (n = 11). When present, with or without other indications, anemia was the main indication for GI endoscopy, so that 89% (n = 49) of the endoscopic procedures were performed because of anemia in order of priority. Anemia was explored because red cell transfusions were needed (n = 36), and/or because anemia was considered to worsen a preexisting chronic heart failure (49%, n = 27).

### 3.5 GI endoscopies: results and complications

GI endoscopies enabled an immediate diagnosis (Table 1) in keeping with the initial symptoms in 64% (n = 35) of the patients. Cancers were the most frequent diagnoses, in 25% (n = 14) of the patients, including colon or rectal cancers in 20% (n = 11), stomach cancers (n = 2), and pancreatic cancer invading the duodenum (n = 1). Cancers represented 40% (n = 14) of all immediate diagnoses (n = 35).

Other significant lesions were gastric or duodenal ulcers/erosions without malignancy in 22% (n = 12) of the patients, esophagitis (n = 3), angiodysplasia (n = 3) and ischemic colitis (n = 2).

Therapeutic modifications as a result of initial GI endoscopy occurred in 94% (n = 33) of the 35 initial diagnoses, including the decision for palliative treatments in 29% (n = 10) of these cases.

Among the 20 patients without a significant GI lesion, 13 had an association between a minimal digestive lesion and antithrombotics.

Regarding the 33 patients with IDA at inclusion, an immediate diagnosis was found in 73% (n = 24), including 30% (n = 10) GI cancers: colon or rectal cancer (n = 9), gastric cancer (n = 1); *DNS*.

Complications following GI endoscopy or anesthesia (Table 1) were noted in 9% (n = 5) of the patients; most of these complications were mild, all of which were delirium (n = 4). One life-threatening complication was recorded: acute heart failure. There was neither colon perforation nor death.

### 3.6 Patient follow-up

#### 3.6.1 Outcome

Average duration of follow-up was 36 ± 24 months. Anemia persisted or recurred in 84% (n = 38) of the patients, with a mean follow-up hemoglobin level of 9.5 ± 1.8 g/dL. Red cell transfusions were performed during follow-up in 20% (n = 9) of the patients.

**Figure 2:**
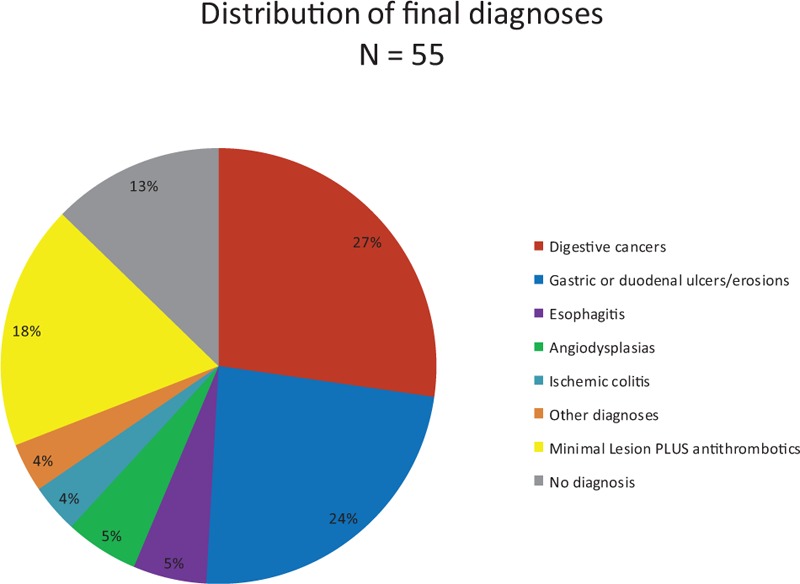
Distribution of final diagnoses.

Thirty-one percent (n = 14) of the patients had further investigations owing to a lack of diagnosis after the initial GI endoscopy. Among these 14 patients, 2 had a late diagnosis explaining initial IDA, always by means of a second-look GI endoscopy: colon cancer (n = 1), and bleeding duodenal ulcer (n = 1), *H pylori*-positive. Cancer was the most frequent diagnosis, with 26% (n = 15) of all patients ultimately diagnosed with cancer (See distribution of final diagnoses in Fig. 2).

Concerning the 13 anemic patients who initially had an association between a minimal lesion and antithrombotics, 77% (n = 10) had a follow-up >12 months without any other diagnosis.

#### 3.6.2 Comparison for survival

Among the 18 deaths, 28% (n = 5) corresponded with the initial indication for GI endoscopy, all from cancers: colon or rectal cancer (n = 3), stomach cancer (n = 1), and pancreatic cancer invading the duodenum (n = 1).

Apart from shorter follow-up, significant differences (Table 2) were found as follows: a higher hemoglobin level at baseline among deceased patients (9.5 ± 1.7 g/dL vs. 8.0 ± 1.7 g/dL; *P* *=* .008); a lower rate of red cell transfusions during follow-up (44% vs. 15%; odds ratio, OR 0.23; 95% confidence interval, CI [0.04–1]; *P* *=* .04); a higher rate of cancer diagnoses (44% vs. 15%; OR 0.23; 95% CI [0.04–1]; *P* *=* *.04*); and more frequent decisions for palliative care (33% vs. 3.7%; OR 0.08; 95% CI [0–0.78]; *P* *=* 0.01).

**Table 2:**
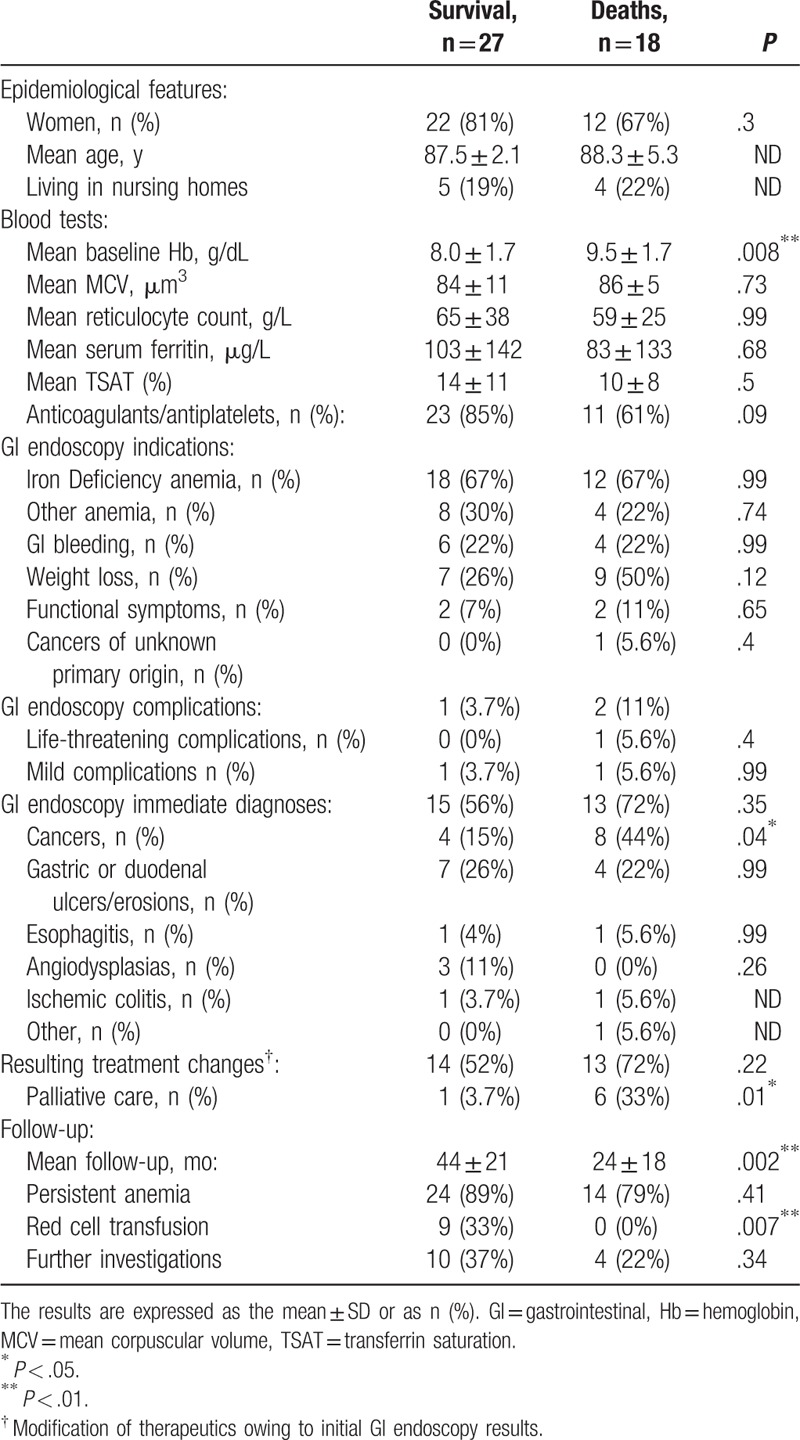
Patients comparison for survival.

#### 3.6.3 Comparison for cancer diagnosis

Fifteen patients were diagnosed with cancer, including 14 early diagnoses and 1 late diagnosis made by a second-look lower GI endoscopy owing to persistent IDA. Compared with patients without cancers (Table 3), cancer patients had a significantly lower reticulocyte count at baseline (45 g/L [interquartile range, IQR 33–53] vs 60 g/L [IQR 49–72]; *P* *=* .02); initial GI endoscopy led to more frequent therapeutic modifications (93% vs. 63%; OR 8.2; 95% CI [1.03–377]; *P* *=* 0.04), including more decisions for palliative care (67% vs. 0%; *P* *<* 0.001); during follow-up, cancer patients received fewer red cell transfusions (0% vs. 28%; *P* *=* 0.02), had fewer further investigations (6.7% vs. 40%; OR 0.11; 95% CI [0–0.87]; *P* *=* .02) but had a higher mortality (53% vs. 25%; OR 3.3; 95% CI [0.9–14]; *P* *=* .05).

**Table 3:**
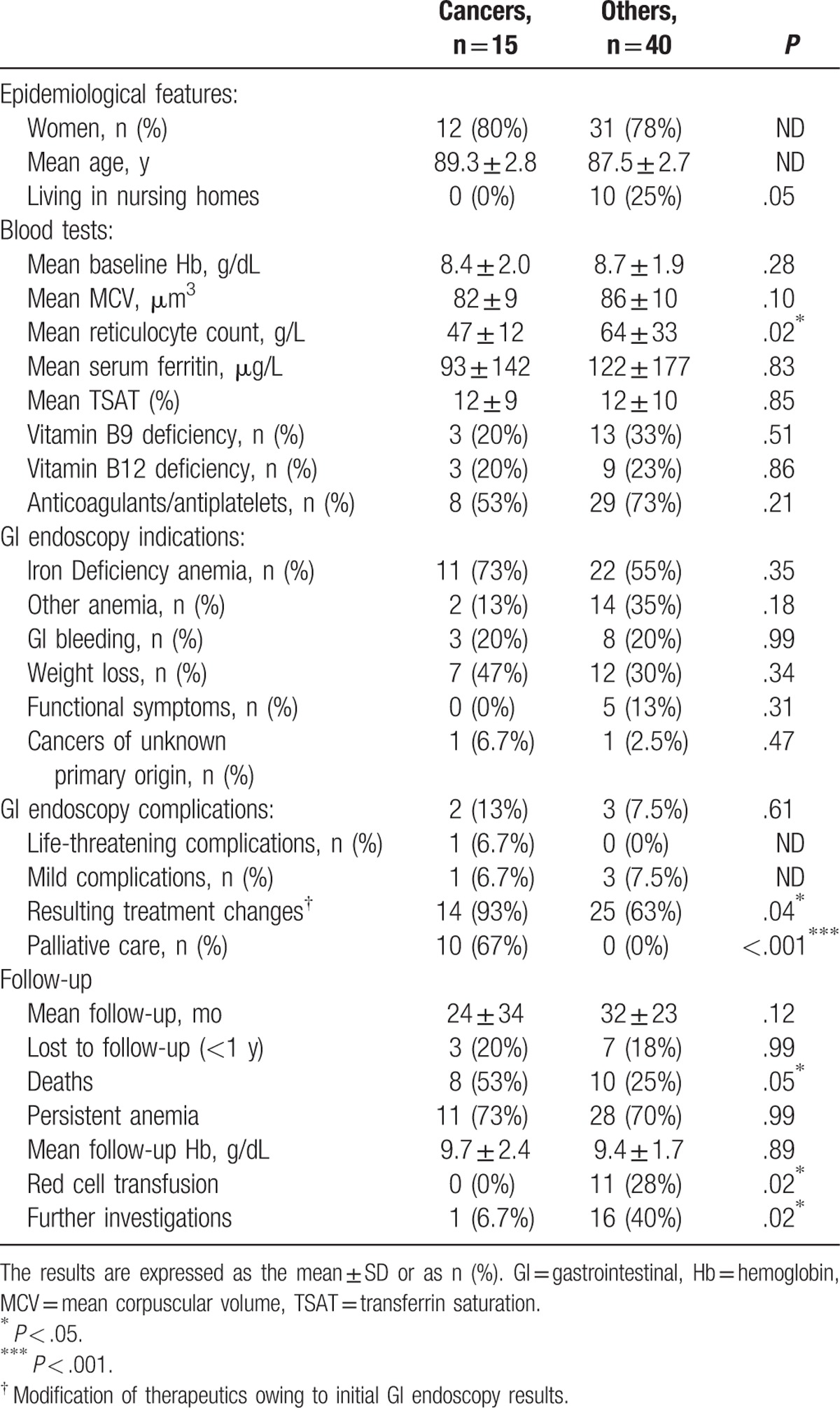
Patients comparison for cancer diagnosis.

The mean follow-up of the 15 patients diagnosed with cancer was 23 ± 34 months. Among these patients, 3 (20%) had surgical treatment immediately after cancer diagnosis and were still relapse-free survivors after 5 years. No patient received chemotherapy.

## 4 Discussion

Our study showed that GI endoscopies at presentation led to an immediate diagnosis in most of patients older than 85 years. More than one-third of the immediate diagnoses were GI cancers. Significant therapeutic modifications were undertaken in 93% of the immediate diagnoses, with 29% of these decisions involving palliative care. During follow-up, patients diagnosed with cancer had significantly fewer red cell transfusions and fewer further investigations. Only 9% of GI endoscopies led to complications, mostly mild. No death was because of GI endoscopy.

The average age of 88 years in our study was the highest in the literature of GI endoscopy in elderly patients.^[18,19]^ Indeed, we aimed to focus on elderly patients with comorbidities. Consequently, we focused on inpatients.

The rate of immediate diagnosis owing to initial upper and lower GI endoscopy, 64%, is similar to that of prior studies of GI investigations of IDA among younger patients,^[15,16,18,20]^ but we found the highest rate of GI cancers. Although previous studies of GI investigations of IDA found 10% to 20% GI cancers among elderly patients,^[19]^ 25% of our patients were immediately diagnosed with GI cancers, representing 40% of the initial diagnoses. An increase in GI cancer incidence with age, reaching 300 per 100,000 at age 85 for colon cancer,^[21]^ is probably the main factor explaining this high rate of GI cancer diagnosis. It is noteworthy that GI endoscopy was often the first tool for cancer diagnosis as only 2 patients were explored by endoscopy following the discovery of metastasis by computed tomography scan.

The diagnostic usefulness of GI endoscopies for patients above the age of 85 was reinforced by the many therapeutic modifications made as a result of associated findings. Remarkably, the outcomes of the patients diagnosed with GI cancers were significantly different from those of other patients, regarding red cell transfusions and the implementation of further investigations during follow-up; both of these interventions occurred less frequently in cases of a cancer diagnosis. Two factors may explain these differences. The primary factor appeared to be the frequent decision to treat with palliative care. The second factor could be the 20% cure rate in those diagnosed with a GI cancer. In both situations, the importance of making the diagnosis of GI cancer, even after 85 years of age, should be highlighted.

Other diagnoses could lead to noninvasive efficient treatments, such as gastric or duodenal ulcers. Besides, the frequency of the association between a minimal digestive lesion and antithrombotics in elderly patients, as showed our former study,^[22]^ could increase the rate of therapeutic modifications, by treating the minimal lesion or by stopping antithrombotics.

Despite older age and a median ASA status of 3, severe complications associated with anesthesia or initial GI endoscopy were rare. No death was reported. These results are similar to previous data on GI endoscopy among patients older than 80 years, reporting <1% severe complications.^[23]^ The median ASA status of three in our population was mostly because of chronic heart failure or coronary heart disease, which seems to be consistent with the high prevalence of cardiovascular diseases over 85, reaching >55%.^[24]^ However, a focus on chronic heart failures in our study shows that the proportion of 51% is higher than the findings of large epidemiological studies, such as the Rotterdam study.^[25,26]^ This large percentage of chronic heart failures could select patients at higher risk of life-threatening complications but does not have a patent influence on our complication rate. It thus reinforces the idea that elderly patients with comorbidities can be explored by a GI endoscopy. Besides, this high proportion of chronic heart failures may be associated epidemiologically with the high rate of anemia in our study, as prevalence of anemia in patients with heart failure is >33% in several studies.^[27–30]^ Moreover, the anemia itself is a comorbidity that can worsen cardiac function^[30]^ and is associated with worse outcomes.^[31–34]^ We also underline the frequent indication of red cell transfusions in our population. Both red cell transfusions and chronic heart failures with concomitant anemia led physician to actively investigate and treat anemia; hence, the frequent indication of GI endoscopy in our study, in spite of old age and ASA status.

In regards to survival, mortality rates reached 40% after 24-month mean survival. Thus, mortality was higher than in the overall 85-year-old population,^[35]^ partially because of the prevalence of chronic heart failures, as outlined above, and because of deaths from GI cancers in 28% of the patients. Cancer diagnoses led to decisions for palliative care in most cases. Moreover, our high death rate was probably associated with anemia, which was present in 89% of our patients. Indeed, anemia itself is associated with mortality.^[36]^

Regarding laboratory tests, hemoglobin level of 8.6 g/dL was less than that in most other studies of endoscopic investigation of IDA^[17,37]^ and was consistent with the lower mean hemoglobin level among patients older than 85, such as in NHANES III.^[6,8]^ Concerning the serum ferritin level used to definite IDA, we chose the threshold of 100 μg/L, adapted for use with elderly patients with comorbidities.^[37–39]^ This higher threshold may have led to the most frequent indication of GI endoscopy being IDA in our population.

### 4.1 Limitations

This study is retrospective, which is the main limitation. However, all eligible patients0 were systematically included and followed by the same physician. We chose to exclude patients with incomplete lower endoscopy to avoid missed colon lesions. This aim of diagnostic detail, and the selection of inpatients older than 85 able to undergo general anesthesia to perform the upper and complete lower GI endoscopy, selected patients presenting a relatively rare situation. Consequently, the sample size of the study was relatively small.

The selection of such patients may have influenced the outcomes of the study. Nevertheless, the high rate of cardiovascular diseases and the median ASA status show that patients with comorbid conditions and frailty were included.

Note that no comprehensive geriatric assessment was available. The selection of elderly patients undergoing GI endoscopy should be based on the Katz ADL scale^[40]^ and the Lawton IADL scale.^[41]^ However, these scales are rarely used in internal medicine units, so they could not be calculated retrospectively for our patients, leading us to use only age to define our study population. Age older than 85 was an objective and broad eligibility criterion, which may have led to the inclusion of patients who might not have been explored by GI endoscopy if following ADL and IADL scales. Whereas ADL and IADL scales may be of importance for therapeutic decisions, our study showed the importance of making a cancer diagnosis, even if subsequently leading to a decision for palliative care.

### 4.2 How to apply these knowledges for routine clinical practice?

An upper and complete lower GI endoscopy must be discussed even in patients >85 years presenting with anemia, especially IDA, GI bleeding, or weight loss from unknown origin. Selected patients with ASA III and comorbidities such as chronic heart failure may also benefit from such a complete GI endoscopic procedure, although a general anesthesia is needed.

## 5 Conclusion

Our results allow us to suggest the potential utility of upper and complete lower GI endoscopy, even in selected patients older than 85 years. Despite frequent decisions to treat with palliative care, the high rate of cancer diagnosis is responsible for substantial therapeutic modifications. Furthermore, severe complications of GI endoscopy remain rare.
